# IMPACT - Integrative Medicine PrimAry Care Trial: protocol for a comparative effectiveness study of the clinical and cost outcomes of an integrative primary care clinic model

**DOI:** 10.1186/1472-6882-14-132

**Published:** 2014-04-07

**Authors:** Patricia M Herman, Sally E Dodds, Melanie D Logue, Ivo Abraham, Rick A Rehfeld, Amy J Grizzle, Terry F Urbine, Randy Horwitz, Robert L Crocker, Victoria H Maizes

**Affiliations:** 1 Health Unit, the RAND Corporation, Santa Monica, CA, USA; 2 Arizona Center for Integrative Medicine, University of Arizona College of Medicine, Tucson, AZ, USA; 3 University of Arizona College of Nursing, Tucson, AZ, USA; 4 Center for Health Outcomes & PharmacoEconomic Research (HOPE), University of Arizona College of Pharmacy, Tucson, AZ, USA

**Keywords:** Integrative medicine, Observational study, Study protocol, Fidelity monitoring, Implementation fidelity, Complex interventions, Comparative effectiveness research, Healthcare utilization, Cost outcomes, Health services research

## Abstract

**Background:**

Integrative medicine (IM) is a patient-centered, healing-oriented clinical paradigm that explicitly includes all appropriate therapeutic approaches whether they originate in conventional or complementary medicine (CM). While there is some evidence for the clinical and cost-effectiveness of IM practice models, the existing evidence base for IM depends largely on studies of individual CM therapies. This may in part be due to the methodological challenges inherent in evaluating a complex intervention (i.e., many interacting components applied flexibly and with tailoring) such as IM.

**Methods/Design:**

This study will use a combination of observational quantitative and qualitative methods to rigorously measure the health and healthcare utilization outcomes of the University of Arizona Integrative Health Center (UAIHC), an IM adult primary care clinic in Phoenix, Arizona. There are four groups of study participants. The primary group consists of clinic patients for whom clinical and cost outcomes will be tracked indicating the impact of the UAIHC clinic (n = 500). In addition to comparing outcomes pre/post clinic enrollment, where possible, these outcomes will be compared to those of two matched control groups, and for some self-report measures, to regional and national data. The second and third study groups consist of clinic patients (n = 180) and clinic personnel (n = 15-20) from whom fidelity data (i.e., data indicating the extent to which the IM practice model was implemented as planned) will be collected. These data will be analyzed to determine the exact nature of the intervention as implemented and to provide covariates to the outcomes analyses as the clinic evolves. The fourth group is made up of patients (n = 8) whose path through the clinic will be studied in detail using qualitative (periodic semi-structured interviews) methods. These data will be used to develop hypotheses regarding how the clinic works.

**Discussion:**

The US health care system needs new models of care that are more patient-centered and empower patients to make positive lifestyle changes. These models have the potential to reduce the burden of chronic disease, lower the cost of healthcare, and offer a sustainable financial paradigm for our nation. This protocol has been designed to test whether the UAIHC can achieve this potential.

**Trial registration:**

Clinical Trials.gov NCT01785485.

## Background

This article describes the protocol for a two-year study of the health and economic impact of a primary care integrative medicine (IM) clinic. IM is defined as a health promoting, prevention-oriented clinical paradigm that explicitly includes conventional medical treatment together with the use of complementary medicine (CM) modalities that have sufficient evidence and safety for use [1-5]. Practice models in IM are emerging [6], but most of the models provide consultative or specialty care; few provide primary care. There are also few methodologically valid health services research studies on the clinical and cost effectiveness of IM practice models, particularly for primary care [7-12].

The evaluation of a IM primary care model presents a number of methodological challenges. Some are the result of the evolving nature of the concept of IM [12], and most have been discussed in the literature to illustrate the complex needs of CM research [13-20]. In general, these challenges apply to research involving any complex intervention (e.g., a new care delivery model) [21-24]. Each challenge is discussed below accompanied by recommended solutions.

### Complex, individualized intervention

Although IM is defined as both a clinical treatment paradigm and a practice organization model, the existing evidence base for IM depends largely on studies of the individual components [7,8,10,14,25]. While IM combines conventional and evidence-based CM modalities (having practitioners who can offer these CM modalities is an important aspect of the practice organization model), it is also a larger clinical paradigm for a process of whole person, patient-centered, healthcare that embraces the innate healing capacity of the human organism and attends to the importance of lifestyle [2,26,27]. Whether seen as a clinical paradigm or as a practice model, IM meets the definition of a complex intervention. It consists of a number of interacting components that make up and affect care, and these components are applied with a large degree of flexibility and tailoring [28]. To create an evidence base for IM itself, studies that characterize and evaluate the outcome of intact IM clinic practices are needed.

Several researchers have recommended that complex interventions simply be studied as "black boxes” [14,16,19,20]; as interventions whose outputs can be measured, but whose internal mechanisms are unknown. Since subjects can be randomized to one black box or another, no new methods are required. Others recommend that the boxes be "unpacked”—that every element in the intervention be defined and its outcomes measured [29,30]. The "black box" approach offers no assurance that the box contents are a valid representation of the intervention; however, given the number of different treatments and potential diagnoses, complete "unpacking" may prove an insurmountable measurement, analysis, and interpretation challenge. The concept of implementation fidelity offers a balance between these two extremes. Outcomes are still measured for, and attributed to, the intervention as a whole (the “box”). However, the contents of the box are defined and measured to document what was actually delivered—i.e., what the box contains.

When a study compares two drugs (or a drug to placebo), all else is held (or assumed to be) equal – in part through randomization, and in part through strict study protocols, including participant and practitioner blinding. As both comparison groups are being treated within the same practice model (e.g., conventional primary care), little, if any, effort is given to defining that model. Faithfulness to the intended intervention, defined as Intervention implementation fidelity, is ensured through monitoring adherence to protocols. The objective is to ensure that the only difference between groups is receipt of the drug under study [22].

In contrast, when two practice models are compared in a study, very little can be held equal. The study protocol is by necessity less prescriptive as to what is delivered, when, how, and by whom. The constraints placed upon possible interventions are loosened to allow practitioner autonomy in individualizing patient care. Not only may different treatments be offered, but different practitioners with different training may diagnose and then prescribe those treatments with different clinical goals and under different philosophies of care. Some method of defining the practice models is needed to capture and measure differences to ensure that interventions are implemented as intended, and to know what is actually being compared.

The concept of intervention implementation fidelity emerged in the 1960s in the field of psychotherapy when early attempts to sort out design and interpretive problems in outcomes studies proved impossible. For an overview of fidelity and its measurement, see Bond et al. [31]. Since the 1960s, including measures of intervention fidelity has become a standard requirement for research in a number of applied fields, including medicine [21,22,24,28,32,33]. Fidelity monitoring is important for the interpretation of study results (e.g., avoidance of Type III error – erroneously concluding that an intervention had no impact when it was actually not implemented), and for replication [22,23,32]. Fidelity measures can also be used as covariates in the analyses to determine treatment effect and avoid invalid statistical conclusions [24].

Assessment of implementation fidelity will help with defining ‘what was measured’ in complex interventions. However, to understand the ‘why it happened’ causal effects of IM, a number of researchers have recommended the use of mixed methods—e.g., including qualitative methods in the study design [13,15,20,21,34]. Randomized controlled trials (RCTs), even when coupled with advanced quantitative methods, cannot explain complex causal relationships [20,21]. Well-designed multi-method studies allow a deeper understanding of the mechanisms that might be at work in the intervention [21].

### Whole person outcomes as markers of effectiveness

IM’s focus on treating the whole person, is believed to have outcomes beyond those associated with treatment of the targeted condition [25,35]. The emphasis on self-care, the benefits of a good patient-practitioner relationship, and the promotion of the body’s self-healing capacity are all believed to result in enhanced health [35]. In parallel to the concept of treating the whole person is the concept of using a whole system of healthcare services delivery. In IM this includes a focus on the patient-provider relationship and a full range of potential treatments. Together these result in the need to measure outcomes across a number of dimensions (e.g., spiritual, social, physical, mental, emotional) [29,36]. The Federal Coordinating Council for Comparative Effectiveness Research (CER) has stressed the importance of “*assessing a comprehensive array of health-related outcomes…”*[37] to provide the information that patients, clinicians and policy makers need to make good healthcare decisions.

### Patient preferences and the infeasibility of randomization

Patients are rarely neutral about IM. They typically indicate strong preferences for or against this approach to healthcare, which makes it hard to accrue patients in randomized trials [14]. In the absence of the ability to randomize appropriately, observational approaches are recommended [16,18]. Observational studies are also recommended for complex systems [19] and to capture actual practice [29]. When well-designed, they have been found to produce results comparable to RCTs, neither over- nor underestimating treatment effects [38-40]. CER methods allow for studies of all types of health-related interventions, including delivery systems [21], and recognize observational studies as valid designs [41-43].

The protocol described in this article addresses the clinical and economic evaluation of an IM primary care clinic. Although the outcomes and processes of care of this particular clinic, as defined in its business plan, are being evaluated, it is anticipated the findings from this study will contribute to IM health services research in general. Because IM practice is a complex intervention, the study design includes fidelity monitoring and evaluation, and an observational design that includes qualitative data collection and analysis. And because IM practice offers whole-person care, a broad range of outcomes are being measured.

## Methods/Design

This two-year study is Phase II of a two-phase, three-year project. The purpose of Phase I was to design the protocol for the Phase II study, including clarifying the design of the IM clinic model to be tested and identifying the measures to use for outcomes and to monitor treatment fidelity. The purpose of Phase II is to conduct a prospective outcomes evaluation of the clinical and cost effectiveness of an IM professional practice model for adult primary care.

### Study design

A combination of observational quantitative and qualitative study designs will be used to measure the health and healthcare utilization outcomes of a primary care IM clinical model. Where possible, the clinical and economic impacts seen in this IM model will be compared to data available from one or more comparison groups (see discussion of comparison groups below). When comparison group data are not available for outcomes —e.g., for most of the self-report data and for the medical records data—baseline values for clinic participants will be compared to those measured during and after treatment. Table 1 shows the types of comparisons planned for each outcome data category for the observational study.

**Table 1:** Outcomes measured for the UAIHC

	**Frequency of measurement**	**Pre/post comparisons**	**Comparisons against control group(s)**
Healthcare utilization and costs	One year prior and one year post-study enrollment	X	X^*^
Diet quality	BL, 3, 6, and 12 months	X	X^**^
Exercise	BL, 3, 6, and 12 months	X	X^**^
Patient satisfaction	3, 6, and 12 months	X	X^***^
Self-report health outcomes	BL, 3, 6, and 12 months	X	
• Self-efficacy			
• Stress			
• Sleep quality			
• Pain			
• Fatigue			
• Depression			
• Anxiety			
• Health-related quality of life			
• Well being			
• Work productivity			
• Condition-specific			
• outcomes			
Medical records data	3, 6, and 12 months	X	

BL = baseline (clinic enrollment).

^*^Primary outcome; comparison is to two matched control groups.

^**^Comparison here is to national (and where available statewide) averages from the Center for Disease Control and Prevention’s Behavioral Risk Factor Surveillance System (BRFSS) survey [51].

^***^Comparison here is to national and regional norms using the Consumer Assessment of Healthcare Providers and Systems (CAHPS) Adult Visit Survey 2.0 data [50].

Because the IM professional practice model is a complex intervention, implementation fidelity will be assessed, in addition to clinical and economic outcomes. Fidelity data (gathered on patients’ and practitioners’ experiences and from medical record data) will serve four purposes. First, these data will be used as a measure of intervention integrity—i.e., was the intervention/practice delivered as intended. Second, because integrative primary care has not previously been defined and evaluated, and since the clinic under study is new, and thus continuously developing and modifying operational and treatment protocols and procedures, these fidelity data will also describe what happened. Third, data on operations and how they may have evolved over time are important as potential covariates in the analysis of outcomes, for proper interpretation of results, and for external validity and replication. Finally, fidelity data provide quality control feedback to clinic staff as to how the IM clinic model is being implemented by staff and perceived by patients. This will allow for quality improvements of the IM primary care clinic over time.

This study received the approval of the University of Arizona Institutional Review Board, and is registered in clinicaltrials.gov (NCT01785485).

### Setting and Participants

Data used in the evaluation will be gathered from eligible and consenting participants who are also patients enrolled at the University of Arizona Integrative Health Center (UAIHC), an integrative medicine adult primary care clinic in Phoenix, Arizona. Operated in conjunction with District Medical Group, Inc., UAIHC is financed through insurance reimbursements and a patient membership fee structure. At present, two major employers in the Phoenix area pay a portion of the membership fee for their employees who elect UAIHC as their primary care provider. There are four groups of study participants. The first (and main) group consists of eligible participants from the clinic patient population for whom clinical and cost outcomes will be tracked indicating the impact of the UAIHC clinic (n = 500). The second and third groups consist of clinic patients (n = 180) and clinic personnel (n = 15-20) from whom fidelity data will be collected. The fourth group is made up of the patients (n = 8) whose path through the clinic will be studied in detail using qualitative methods. The clinic patients involved in the outcomes study sample may or may not be included in the fidelity patient sample, and vice versa. However, the qualitative data sample is expected to be a subset of the outcomes sample.

### Outcomes sample

Only patients who are members at UAIHC and who meet the inclusion/exclusion criteria are eligible to enroll in this study. Participation in the study will continue for at least one year after enrollment. It is anticipated that at least 500 patients will consent to participation during the first nine months of the study. Our primary outcome for our main analysis is change in total costs (see the Analysis section below). Using the standard deviation seen in a set of healthcare utilization data from a related group, a sample size of 200 patients should be sufficient to detect a small effect size (Cohen’s d = .20) difference in total costs at a .80 power using an alpha of .05. Therefore, our expected sample size of 500 may also allow for some subgroup analysis. Inclusion criteria: 1) Enrollment in primary care (members) at UAIHC; and, 2) adults ages 18 or older. Exclusion criteria: 1) currently pregnant at time of recruitment; 2) patients who attend the UAIHC clinic as “consultation-only” patients; and, 3) significant cognitive impairment to the extent that the individual is unable to understand the consent and respond to questionnaires.

All new patients will be advised by their primary care physician that they may be eligible to participate in a study to evaluate the effectiveness of the UAHIC. Prior to checkout, patients have an exit session with the University of Arizona (UA) Study Coordinator. During this session (or if necessary, through a follow-up phone call), the Study Coordinator will explain the nature of the study and make a determination of study eligibility, and if appropriate, consent the patient. Patients who joined the UAIHC before study start (July 2013) will also have the opportunity to participate. The study will be explained at their first UAIHC visit after study start.

### Fidelity patient sample

Participants in the fidelity study will include both UAIHC patients and members of the UAIHC clinic team. On fidelity assessment sample days (a single randomly chosen day of the last week of the data collection month; see data collection procedures below), participants will be recruited and enrolled from all patients who enter the UAIHC waiting room. It is anticipated that approximately 180 patients will consent to participation across the two-year study period. The inclusion/exclusion criteria for this sample are identical to those used for the outcomes sample with the exception that patients who are currently pregnant will be allowed to participate in this sample.

At check in on the fidelity data collection day, front desk staff will make a printed handout available describing a “How is UAIHC Doing?” evaluation, and asking all patients seen that day to provide feedback through an online questionnaire after that day’s visit. Interested patients will supply their e-mail address and check a box on the printed handout expressing permission to receive the online questionnaire. The UA Study Coordinator will send all interested patients an e-mail with instructions and a link to the secure URL address for the fidelity informed consent form and the Web-based questionnaire. Patients preferring to complete the questionnaire on paper will have the opportunity to receive a hard copy from the front desk (or by mail) with instructions to place completed questionnaires in a secure box at the clinic (or to return them using the enclosed self-addressed stamped envelope). Digitally-signing the informed consent form, or returning a signed paper-based consent form will constitute completion of the informed consent process for fidelity. Patients who complete the questionnaire within 10 days will receive a $10 gift card.

### Fidelity clinic personnel sample

The UA Study Coordinator will enroll UAIHC personnel at study start by sending an e-mail with a link to the secure URL address and password information for the informed consent form. Assessments will be on the same schedule as for patient fidelity (see Data collection below). A total of 15–20 clinic personnel (the entire UAIHC staff) will be enrolled in the fidelity study. The same consent process and incentive will be used as for the fidelity patient sample.

### Qualitative/case study sample

The qualitative study sample will be a subset of the outcomes sample. In addition to the inclusion and exclusion criteria of the outcomes sample subjects will consist of members who joined the study during its first three months. The qualitative study will be introduced to patients by the UA Study Coordinator during the same meeting in which the outcomes study is discussed. Participation will require an additional consent, and will entail a 20–30 minute semi-structured, taped interview immediately following each regular outcome data collection points.

### Intervention

The IM professional practice model for primary care utilizes an inter-professional, physician-led, integrative clinical team to provide patient-centered care. UAIHC services include conventional medical care, lifestyle interventions, and evidence-based CM interventions. In particular, services include longer visits with providers, a commitment to health, a partnership agreement with the health care team, increased access via telephone and email in addition to in-person visits and classes, and a wider range of services including nutritional counseling, acupuncture, chiropractic care, stress-reducing mind-body approaches, and health coaching. In addition to individual appointments, group visits and health promotion classes are offered (e.g., nutrition, yoga, meditation). Emphasis is placed on motivating and supporting lifestyle behavior change. Although patients with a wide variety of diagnoses will be eligible and included in the study, outcomes for patients with the following five conditions (low back pain, fibromyalgia, other musculoskeletal conditions, metabolic syndrome and diabetes, and cardiovascular disease) will be analyzed separately where possible.

The UAIHC business model uses a hybrid financing approach that utilizes usual insurance reimbursements supplemented by a patient membership fee. The membership fee covers a certain number of CM practitioner visits, health coaching visits, and classes (a “class” may comprise a series of individual sessions). Three fee tiers have been established: 1) basic bundle (5 visits with the patient’s choice of CM practitioners, 2 visits with a health coach, and 2 groups or classes); 2) core (10 visits with the CM practitioners of choice, 4 health coach visits, and 4 groups or classes); and, 3) expanded (20 visits with CM practitioners, 8 visits with a health coach, and 8 groups or classes). For some members, this fee is partially offset by collaborating employers who offer UAIHC as an employee health benefit option. (Grant funding supports the costs of this study for those participants employed with these collaborators).

According to its business plan, the IM primary care practice model will follow IM principles [44] as well as the principles of primary care [45,46] and the patient-centered medical home [47]. More detail on the IM primary care practice model is presented in Dodds et al [48].

### Comparison group(s)

Data from UAIHC patients will be compared, where possible, to those of patients seen in other clinics. The primary outcome, total healthcare utilization, will be obtained from healthcare claims data for patients whose employers have given access to those data. For these patients, matched comparison groups will be developed using two different matching algorithms applied to de-identified claims data for non-UAIHC patients. One will use a straight matching algorithm based on age and gender, and the other will use propensity scores to match [49]. At least one of the participating employers also has employee lab values available. Where these data are available, the same matched comparison groups will be used to compare changes in key lab values seen across groups. Finally, several of the self-report measures have national and regional comparisons available. The measures of patient experiences and patient satisfaction are drawn from the Consumer Assessment of Healthcare Providers and Systems (CAHPS) Adult Visit Survey 2.0 [50]. The CAHPS website contains data on the results of these questions nationally, by region, and by physician specialty. The results seen for UAIHC participants will be compared to those seen by all providers nationally, by all providers in the West region, and by family practice doctors nationally. The measures of diet and exercise quality come from the Center for Disease Control and Prevention’s Behavioral Risk Factor Surveillance System survey [51]. National and statewide averages are available for these items. Unfortunately, no other secondary data sources are available for comparisons. For all other outcomes, UAIHC participants will act as their own controls by comparing their outcome measures over time to baseline.

### Data Collection

For the outcomes evaluation, data will be collected from patient charts and self-reports at four intervals—baseline (initial clinic visit), and at 3-, 6-, and 12-month follow up periods. Participants will receive a reminder email one week before each data collection due date. Baseline data will be due within one week of enrollment. In the email they will be directed to a secure website where they can access and complete the outcomes data collection instrument. If participants have not entered their data within 3 days of their due date, they will receive another reminder email, and those who have not entered their data within another 3 days of their due date will receive a call from the UA Study Coordinator. Healthcare utilization and cost data from healthcare claims will be obtained from their employer with study participant consent. These data will be collected for one year before and one year after clinic enrollment approximately 3 months after the 12-month follow up period to allow time for all incurred claims to be recorded. De-identified claims data for the comparison groups will be collected for the same period of time with “baseline” being defined by the average start date of study participants.

Because the intervention may change over time as clinic operations start up and mature [21], fidelity monitoring data will be collected monthly from study start for 6 months, then quarterly for 6 months, and then semi-annually. Patient-reported fidelity data will be gathered on a single randomly chosen day of the last week of the data collection month from all patients seen at the UAIHC clinic that day. This day will be selected by the UA research team using a simple computer generated randomization scheme and delivered to the UA Study Coordinator the week before the data collection week. A self-administered patient version of the “How is UAIHC doing?” questionnaire will be used for patients. For clinic personnel, fidelity data will be collected during the last week of each fidelity data collection month using an online self-administered practitioner and staff version of the “How is UAIHC doing?” questionnaire. Data from audits of randomly selected, de-identified patient charts and of administrative records will also be used to assess indicators of model implementation.

Qualitative data in the form of the results of 20–30 minute semi-structured interviews will be collected by phone or in-person approximately every three months, and at other significant points in time if the patient agrees—e.g., if the patient chooses to leave the clinic or there is a dramatic change in the patient’s use of clinic services. The focus is to collect real-time data on what the patient believes is the cause of any changes (or lack of change) seen in their health.

### Outcome measures

Data on clinical and economic outcomes will be collected from three main sources: self-report instruments, chart abstraction, and health plan administrative data (healthcare utilization and, where available, lab values) from participants’ employers. Because integrative medicine approaches treat the whole person, rather than simply targeting a specific disease or symptom, they can have broad impacts on health [25,29,35,36]. Therefore, a broad set of clinical and economic outcomes for the UAIHC will be captured (Table 1). These are each discussed below, starting with the self-report measures. All are collected at baseline (some directly and some as part of patients’ initial clinic intake form), and then 3, 6 and 12 months and semi-annually thereafter, unless otherwise noted.

### Self-report instruments – all patients

In addition to general socio-demographic data and an item on patients’ expectations of the clinic (both asked only at baseline), several factors are captured that could be seen as overall determinants of health and/or intermediate outcomes: diet, exercise, self-efficacy, stress, sleep and patient satisfaction. In addition, a number of specific (pain, fatigue, depression and anxiety) and global (quality of life, well-being, and work productivity) outcomes are captured. Each is described briefly below. It is estimated that it will take patients 15–20 minutes to complete these instruments.

• Diet quality. Diet quality is assessed by the Fruits and Vegetables module of the Behavioral Risk Factor Surveillance System [51]. Six items assess the type of fruits, vegetables, and legumes eaten in the past month and their frequency.

• Exercise & Physical Activity. Physical activity is assessed by the Exercise/Physical Activity module of the Behavioral Risk Factor Surveillance System [51]. Eight items assess the type, frequency, and duration of exercise/physical activity in the past month.

• Health self-efficacy. The Patient Activation Measure (PAM) is a 13-item self-assessment of an individual’s knowledge, skill, willingness, and confidence in managing their health and health care (activation) [52,53]. Scores stratify patients into four levels of health care activation.

• Stress. Several aspects of stress are captured. The Perceived Stress Scale (PSS) [54] is a brief measure that assesses the degree to which situations in one’s life are appraised as stressful in a global manner. Items tap how unpredictable, uncontrollable, and overloaded respondents find the stress in their lives to be. Two single items, found to strongly correlate with established multi-item measures, are used to tap the psychological response to stress and the amount of stress experienced in the past year [55].

• Sleep quality. Sleep quality is assessed by the 9-item Pittsburgh Sleep Quality Index (PSQI) [56]. The PSQI measures the quality and patterns of sleep over the past 1 month. It differentiates “poor” from “good” sleep by inquiring about seven areas: 1) subjective sleep quality, 2) sleep latency, 3) sleep duration, 4) habitual sleep efficiency, 5) sleep disturbances, 6) use of sleeping medication, and 7) daytime dysfunction.

• Patient satisfaction. The 2-item measure of patient satisfaction is from the Consumer Assessment of Healthcare Providers and Systems (CAHPS) Survey 2.0 Adult visit version [50,57]. These items are not included at baseline.

• Pain (global). Global rating of pain is assessed by a single-item pain visual analogue scale (*pVAS)*[58]. It measures severity of pain over the past week.

• Fatigue. Both a global rating and specific symptoms of fatigue are captured. Global rating of fatigue is assessed by a single-item fatigue visual analogue scale (*fVAS)*. It measures the severity of fatigue over the past week. The 9-item Fatigue Severity Scale (FSS) [59] assesses how fatigue affects motivation, exercise, physical functioning, carrying out duties, and interfering with work, family, or social life in the past 1 week.

• Depression. The 2-item ultra-brief Patient Health Questionnaire (PHQ-2) [60] is used to assess core symptoms of major depression. The items ask how often over the last 2 weeks participants were bothered by 1) having little interest or pleasure in doing things (anhedonia), and 2) feeling down, depressed, or hopeless (depressed mood). Scores of ≥ 3 have a sensitivity of 83 % and a specificity of 92 % for major depression. Patients with scores of ≥ 3 will also receive the full PHQ-9 (below).

• Generalized Anxiety Disorder. The 2-item ultra-brief Generalized Anxiety Disorder questionnaire (GAD-2) [61] is used to assess core symptoms of anxiety disorder. The items ask patients how often over the last 2 weeks they were bothered by 1) feeling nervous, anxious, or on edge and 2) not being able to stop or control worrying. Scores of ≥ 3 have a sensitivity of 86 % and a specificity of 83 % for Generalized Anxiety Disorder. Patients with scores of ≥ 3 will also receive the full GAD-7 below.

• Health-related Quality of Life. Health-related quality of life is assessed by the 12-Item Short Form Medical Outcomes Survey (SF-12) [62]. The SF-12 is a general measure of health status and well-being that assesses physical functioning, role limitations due to physical health, bodily pain, general health, energy/fatigue, social functioning, and role limitations due to emotional problems and mental health in the past 4 weeks.

• Psychological Well-being. Psychological well-being is assessed by the 5-item World Health Organization Well-Being Index (WHO-5) [63]. The WHO-5 assesses subjective quality of life as a dimension separate from social disability and asks about dimensions such as positive mood, vitality, and interest in life in the past 2 weeks.

• Health-related work productivity (absenteeism/presenteeism). Health-related work productivity is assessed by the 6-item Work Productivity and Activity Impairment questionnaire (WPAI) [64]. The WPAI is a self-administered instrument to assess the impact of disease on absenteeism (work time missed), presenteeism (reduced on-the-job effectiveness), and impairment in daily activity.

• Healthcare utilization. Four items based on Ritter et al. (2001) [65] ask about use of healthcare services in the prior period—from primary care providers outside of UAIHC, from specialists, from emergency rooms or urgent care centers, and overnight hospital stays.

### Self-report instruments – condition specific

For participants diagnosed by UAIHC with any of the identified study clinical conditions (low back pain, fibromyalgia, depression and anxiety), one or more of the following questionnaires will be added:

• Fibromyalgia Impact Questionnaire Revised - FIQR. The 21-item FIQR is a patient report of symptoms associated with fibromyalgia in three domains: physical functioning (9 items), overall impact (2 items), and symptoms (10 items) [66].

• Roland-Morris Disability Questionnaire – RMDQ. The RMDQ is a patient report of physical disability due to low back pain (Roland & Morris, 1983). The RMDQ contains 24 yes/no items focused on limitations in walking, bending over, sitting, lying down, dressing, sleeping, self-care, and daily activities.

• Patient Health Questionnaire 9 - PHQ-9. To assess clinical depression, the PHQ-9 [67,68] will be used with patients if the PHQ-2 screening score is ≥ 3.

• Generalized Anxiety Disorder 7 - GAD-7. When scores on the GAD-2 exceed the cut point of ≥ 3 on the New Patient Intake Form, the 7-item GAD-7 will be administered [69].

### Data collected from medical records

These data will be collected at 3, 6, and 12 months. A medical records abstraction form will be used to collect the following from the patient chart for the period since the last outcomes data collection point. The results of any of the following lab tests will be captured: fasting glucose, lipid panel, thyroid stimulating hormone (TSH), glycosylated hemoglobin (HbA1c), inflammatory markers (erythrocyte sedimentation rate and high-sensitivity C-reactive protein), vitamin D status, and measures of anemia (hemoglobin, hematocrit, and mean corpuscular volume). Also captured will be each patient’s diagnosis(es), systolic and diastolic blood pressure, heart and respiratory rates, weight, waist circumference, body fat composition, smoking status, and whether the patient is on hypoglycemic, dyslipidemia, or hypertension medication. Composite scores (10-year cardiovascular disease (CVD) event risk [70], 10-year CVD mortality risk [71], and metabolic syndrome diagnosis [72]) will be captured from the chart when recorded, or will be calculated. Forced vital capacity and forced expiratory volume will be captured for participants with pulmonary disease.

### Health Insurance Claims Data

Annually, the UA Study Coordinator will create a coded, numeric list of study participants, who are employees or adult dependents of participating employer groups, to send to their health plan. This list will enable the health plan to generate and return a file of otherwise de-identified health claims data. Study participants will be identifiable (a partial waiver of protected health information data was granted by the UA Institutional Review Board) so that healthcare utilization data can be matched to their other study data, and the remainder of the file will be used to generate the matched control groups. The initial data request will cover one year prior and one year after clinic enrollment for study participants, and a comparable 2-year period for non-participants.

### Fidelity monitoring measures

#### Patient feedback questionnaire

Patient experiences of the various dimensions of integrative medical care will be assessed for their same-day care by a 51-item “How is UAIHC doing?” Patient Version questionnaire developed for the fidelity study. Dimensions of the questionnaire include access to care, whole person care, promotion of self-care and wellness, practitioner communication style, shared decision-making, trust in the practitioner, perceived practitioner empathy, perceived health partnership, and socio-demographic information. Items were derived from established measures of primary care quality: the Consumer Assessment of Healthcare Providers and Systems (CAHPS) Surveys 2.0 Adult visit and Patient-Centered Medical Home versions [50,57], the Ambulatory Care Experiences Survey (ACES) [73], and the Consultation and Relational Empathy measure (CARE) [74]. Several items were developed for the study to tap patient experiences of IM care not assessed in existing instruments. It is estimated that it will take 10–15 minutes for patients to complete this questionnaire.

### Patient chart extraction

A number of items will be extracted from a random sample of 10 de-identified patient charts at the end of each fidelity measurement month for patients seen during that month. The clinic staff will generate a list of the medical record numbers of all enrolled patients seen during the last month and report the total number (N) to the research team. The research team will use a simple computer-based randomization scheme to create an equal length list consisting of “select” and “don’t select” statements. Clinic staff will then match these lists and pull and de-identify those charts whose medical record numbers match up with the “select” statements. The data to be extracted by the research team are intended to assess IM whole person care (e.g., a review of systems beyond those typically assessed in conventional care including domains such as spirituality, social support, family and work stressors, etc.), the patient-practitioner partnership agreement, the mix of conventional and CM therapies used, and lifestyle recommendations over the past month. Visit lengths are also recorded.

### Practitioner experiences survey

Practitioner experiences working in the UAIHC will be assessed using the 27-item “How is UAIHC doing?” Practitioner Version questionnaire developed for the fidelity study. Dimensions of the questionnaire assess the team care climate of the clinic, practitioners’ stress and self-care practices, and practitioners’ feedback on how practicing in the UAIHC model may affect their clinical care. The Team Climate Inventory (TCI) [75], a single-item on practitioner burnout [76], as well as additional individual items to account for IM principles will be administered. It is estimated that it will take 10–15 minutes for staff to complete this questionnaire.

### Qualitative/case study data

The patients who have been selected for and agreed to case study interviews will participate in a 20–30 minute semi-structured interview immediately after each of the outcomes data collection points. The patient’s outcome results over time will be available to the interviewer, and the interview will focus on the patient’s experience of care at UAIHC and what the patient believes has contributed to any health changes seen in the course of treatment. These interviews will be either done by phone or in person, and will be audiotaped and transcribed.

### Analysis

Five sets of inter-related analyses are planned for these data. The first will be a descriptive study based on the demographics and baseline values of the first patients (e.g., first 200) who join the study. The purpose of this study and report is to document the characteristics, diagnoses, symptoms and expectations of those patients who choose to enroll in this integrative primary care clinic. Where possible, patient characteristics, diagnoses and symptoms will be compared to those seen nationally and statewide in primary care using data from the National Ambulatory Medical Care Survey (NAMCS) [77]. Basic descriptive statistics will be used. When comparisons are possible, will utilize *t* tests for independent samples for continuous data, and χ^2^ tests for frequencies.

The second analysis will use the fidelity monitoring data to describe clinic operations and to describe how this IM primary care clinic developed over time. Since these are measures of the intervention itself (the clinic), by design the patient fidelity sample measurements over time will not be taken on the same individuals. Therefore, data will be analyzed using simple descriptive statistics. The results will also be examined for trends using regression analysis with time (either continuous or as a set of dummy variables) as the main explanatory variable, and significant *t* tests for the time coefficient(s) as an indication of significant change over time in clinic operations. Components of the clinic model that change significantly over time and/or that contribute to the outcomes will be considered as covariates in the two outcomes studies described below [24].

The third analysis will involve data from the case study participants. The narrative data from the transcribed, audiotaped semi-structured interviews will be content analyzed using dedicated qualitative data analysis/text management software (*Atlas-ti*), and key concepts will be categorized into salient themes and patterns. Those results will be related to each participant’s reported and recorded outcomes to look for insights into clinic impacts and operations.

The fourth and fifth analyses will be the main outcome studies. The fourth study will be a prospective cohort study of the changes in outcomes from baseline to one year for all patients in the outcomes sample. The primary outcome for this analysis will be health-related quality of life measured in three ways (SF-6D [78], and the physical and mental component summary scores, all derived from the SF-12), with the other outcomes as secondary measures. The main comparison will be baseline to 12-month outcomes and the univariate analyses will use paired *t* tests for continuous data, and use the McNemar test for frequencies [79]. The data will also be examined for patterns of change over time in key outcomes, and the determinants of those patterns, using hierarchical linear modeling (HLM) techniques [80-82]. Fidelity measures with significant changes over time will be used as covariates [24] in these models, as well as other explanatory variables such as demographics, participants’ membership level and baseline diagnoses. If participant numbers for each of the five targeted condition allow, condition-specific outcomes will be examined for each. These same analyses will be applied to other secondary outcomes such as CVD event [70] and mortality [71] risk.

The fifth analysis will focus on the subset of patients for which health insurance claims data are available and will utilize a matched comparison design. Prior to analysis all claims data for each individual (treatment and comparison groups) will be aggregated (by summation) into per health plan member per month (PMPM) totals of paid amounts (the amount paid by the health plan to the practitioner) for each of the following cost categories: total, inpatient, outpatient, emergency room, physician, and pharmaceutical claims. Similarly, the numbers of outpatient visits, hospital stays and emergency department visits will also be aggregated for each individual. The primary outcome will be total cost. Two comparison groups from de-identified claims data will be created—one using simple matching (e.g., by age and gender) and one using propensity scores [49]. After the comparison groups have been selected, simple descriptive analyses will show average PMPM costs and healthcare utilization (including standard deviations and other measures of dispersion) pre- and post- enrollment (or an equivalent date for the comparison groups) by gender, age, baseline diagnosis and by treatment and comparison group. Because cost data tends to be highly skewed, average PMPM costs and healthcare utilization pre/post enrollment, and the change in costs and utilization pre/post between the treatment and each of the comparison groups will be reported as mean differences with confidence intervals calculated using bootstrap methods [83,84]. Because of the number of data points for each patient (e.g., up to 12 PMPM values pre-enrollment and 12 post-enrollment), HLM [80-82] will be used to estimate the pattern of change in healthcare utilization and costs over time, and to explore the determinants of those patterns. Fidelity measures with significant changes over time [24], demographics, participants’ membership level and baseline diagnoses will be included as covariates in these models. Finally, changes in costs between groups will be compared to changes in patients’ CVD risk.

Results for the last two analyses will be reported following the Strengthening the Reporting of Observational Studies in Epidemiology (STROBE) guidelines [85]. For all outcomes, the estimated treatment effects will be reported using their original outcome scale and including confidence intervals.

## Discussion

There has been much written about the need for new models of medical care—ones that are more patient-centered, that can empower patients to make positive lifestyle changes, that can lower cost of healthcare, and that offer a sustainable financial model. This study has been designed to test whether an IM primary care practice model (UAIHC) can meet these challenges.

This study will face a number of limitations. For example, some of the self-report measures of fidelity have not been fully tested and validated. The data collection burden on patients might limit enrollment or increase dropout. Also, because this is a new clinic, member enrollment and study adoption rates are unknown and could fall short of predictions. This is especially true for the five targeted conditions; low prevalence will prevent analyses by condition. Obtaining a useable set of claims data is always challenging. Better outcomes (larger effect sizes) may be achieved were factors in the clinic subject to more control (e.g., patient homogeneity, or treatment consistency rather than patient-centered care), or if the clinic had already had time to mature into its final form. Also, at such an early phase of the clinic’s development and management, the methodological rigor required to study this multi-faceted system of care may not be able to yield all of the desired information. Instead, this will be an evaluation of a newly designed and formed IM primary care clinic model in its natural state as it evolves. Some of the data collected during fidelity monitoring will also be useful in improving and optimizing clinic performance. The addition of more qualitative data collection is desirable to more closely examine patient experiences in the clinic and may be possible as funds and time become available.

This protocol is designed to meet the methodological challenges involved in evaluating this complex intervention (IM model of adult primary care) with as much rigor as possible. It is anticipated that its results and the results of anticipated continuous evaluation of the UAIHC will contribute greatly to health services research in integrative medicine.

## Abbreviations

ACES: Ambulatory care experiences survey; CAHPS: Consumer assessment of healthcare providers and systems; CARE: Consultation and relational empathy measure; CER: Comparative effectiveness research; CM: Complementary medicine; CVD: Cardiovascular disease; FIQR: Fibromyalgia impact questionnaire revised; FSS: Fatigue severity scale; fVAS: Fatigue visual analog scale; GAD-2: Generalized anxiety disorder questionnaire ultra-brief 2-item version; GAD-7: Generalized anxiety disorder questionnaire 7-item version; HbA1c: Glycosylated hemoglobin; HLM: Hierarchical linear modeling; IM: Integrative medicine; NAMCS: National ambulatory medical care survey; PAM: Patient activation measure; PHQ-2: Patient health questionnaire ultra-brief 2-item version; PHQ-9: Patient health questionnaire 9-item version; PMPM: Per member per month; PSQI: Pittsburgh sleep quality index; PSS: Perceived stress scale; pVAS: Pain visual analog scale; RCT: Randomized controlled trial; RMDQ: Roland-Morris disability questionnaire; SF-12: 12-Item short form medical outcomes survey; SF-6D: 6-dimentional Health-related quality of life measure based on the short form medical outcomes survey; STROBE: Strengthening the reporting of observational studies in epidemiology guidelines; T4: Total thyroxine; TCI: Team climate inventory; TSH: Thyroid stimulating hormone; UA: University of Arizona; UAIHC: University of Arizona Integrative Health Center; WHO-5: World Health Organization well-being index; WPAI: Work productivity and activity impairment questionnaire.

## Competing interests

SED, RH, RLC and VHM are all employees of the Arizona Center for Integrative Medicine (AzCIM) at the University of Arizona College of Medicine. Although the UAIHC has been sponsored by AzCIM and AzCIM may gain financially from its success, it is unlikely that the publication of this protocol manuscript would cause AzCIM to gain or lose financially. The remaining authors declare that they have no competing interests.

## Authors’ contributions

PMH participated in the conceptualization and design of the study, drafted the manuscript, and incorporated co-author comments. SED participated in the conceptualization, design, and implementation of the study and critical review and revision of manuscript draft. All other authors participated in the design of the study and review of the manuscript. All authors approved the final manuscript for publication.

## Pre-publication history

The pre-publication history for this paper can be accessed here:

http://www.biomedcentral.com/1472-6882/14/132/prepub

## References

[B1] Maizes V Schneider C Bell I Weil A Integrative medical education: development and implementation of a comprehensive curriculum at the University of Arizona Academ Med 2002 77 9 851 860 10.1097/00001888-200209000-00003 12228072

[B2] Boon H Verhoef M O'Hara D Findlay B Majid N Integrative healthcare: arriving at a working definition Alternative therapies in health and medicine 2004 10 5 48 15478786

[B3] About Us: Definition of Integrative Medicine [http://www.imconsortium.org/about/home.html]

[B4] What is Complementary and Alternative Medicine? [http://nccam.nih.gov/health/whatiscam]

[B5] What is integrative medicine? [http://www.bravewell.org/integrative_medicine/what_is_IM/]

[B6] Horrigan B Lewis S Abrams D Pechura C Integrative Medicine in America 2012 The Bravewell Collaborative: Minneapolis, MN

[B7] Deng G Weber W Sood A Kemper KJ Research on integrative healthcare: context and priorities Explore: The Journal of Science and Healing 2010 6 3 143 158 10.1016/j.explore.2010.03.007 20451148

[B8] Deng G Weber W Sood A Kemper K Integrative Healthcare Research: Context and Priorities 2009 Institute of Medicine: Washington, DC

[B9] Khorsan R Coulter ID Crawford C Hsiao AF Systematic review of integrative health care research: randomized control trials, clinical controlled trials, and meta-analysis Evidence-Based Complementary and Alternative Medicine 2011 2010 10.1155/2011/636134 PMC2952316 20953383

[B10] Marcus DM McCullough L An evaluation of the evidence in" evidence-based" integrative medicine programs Academic Medicine 2009 84 9 1229 10.1097/ACM.0b013e3181b185f4 19707062

[B11] Guarneri E Horrigan BJ Pechura CM The efficacy and cost effectiveness of integrative medicine: a review of the medical and corporate literature Explore: The Journal of Science and Healing 2010 6 5 308 10.1016/j.explore.2010.06.012 20832763

[B12] Coulter ID Khorsan R Crawford C Hsiao AF Integrative health care under review 2010 10.1016/j.jmpt.2010.08.007 21109060

[B13] Cassidy CM Social science theory and methods in the study of alternative and complementary medicine The Journal of Alternative and Complementary Medicine 1995 1 1 19 40 10.1089/acm.1995.1.19 9395601

[B14] Nahin RL Straus SE Research into complementary and alternative medicine: problems and potential BMJ 2001 322 7279 161 164 10.1136/bmj.322.7279.161 11159578 PMC1119420

[B15] Mason S Tovey P Long AF Evaluating complementary medicine: methodological challenges of randomised controlled trials BMJ 2002 325 7368 832 834 10.1136/bmj.325.7368.832 12376448 PMC1124333

[B16] Harlan WR Jr New opportunities and proven approaches in complementary and alternative medicine research at the National Institutes of Health J Alternative & Complemen Med 2001 7 1 53 59 10.1089/107555301300004538 11822636

[B17] Gatchel R Maddrey A Clinical outcome research in complementary and alternative medicine: an overview of experimental design and analysis Alternative Ther Health Med 1998 4 5 36 9737030

[B18] Vickers A Cassileth B Ernst E Fisher P Goldman P Jonas W Kang S Lewith G Schulz K Silagy C How should we research unconventional therapies Int J Technol Assess Health Care 1997 13 111 121 10.1017/S0266462300010278 9119619

[B19] Levin JS Glass TA Kushi LH Schuck JR Steele L Jonas WB Quantitative methods in research on complementary and alternative medicine: a methodological manifesto Medical Care 1997 35 11 1079 1094 10.1097/00005650-199711000-00001 9366888

[B20] Vickers A Methodological issues in complementary and alternative medicine research: a personal reflection on 10 years of debate in the United Kingdom J Alternative Complementary Med 1996 2 4 515 524 10.1089/acm.1996.2.515 9395681

[B21] Alexander JA Hearld LR Methods and metrics challenges of delivery-system research Implementation Science 2012 7 1 15 10.1186/1748-5908-7-15 22409885 PMC3317852

[B22] Hasson H Systematic evaluation of implementation fidelity of complex interventions in health and social care Implement Sci 2010 5 1 67 10.1186/1748-5908-5-67 20815872 PMC2942793

[B23] Dumas JE Lynch AM Laughlin JE Phillips Smith E Prinz RJ Promoting intervention fidelity: conceptual issues, methods, and preliminary results from the EARLY ALLIANCE prevention trial Am J Preven Med 2001 20 1 38 47 10.1016/S0749-3797(00)00272-5 11146259

[B24] Santacroce SJ Maccarelli LM Grey M Intervention fidelity Nursing Res 2004 53 1 63 66 10.1097/00006199-200401000-00010 14726779

[B25] Herman PM Evaluating the Economics of Complementary and Integrative Medicine 2012 Samueli Institute: Alexandria, VA 10.7453/gahmj.2013.002 PMC3833528 24416664

[B26] Rees L Weil A Integrated medicine: imbues orthodox medicine with the values of complementary medicine BMJ 2001 322 7279 119 10.1136/bmj.322.7279.119 11159553 PMC1119398

[B27] Maizes V Rakel D Niemiec C Integrative medicine and patient-centered care. In: IOM Summit on Integrative Medicine and the Health of the Public commissioned paper 2009 Institute of Medicine: Washington, DC 10.1016/j.explore.2009.06.008 19733814

[B28] Craig P Dieppe P Macintyre S Michie S Nazareth I Petticrew M Developing and evaluating complex interventions: the new medical research council guidance BMJ 2008 337 10.1136/bmj.a1655 PMC2769032 18824488

[B29] Bell IR Caspi O Schwartz GER Grant KL Gaudet TW Rychener D Maizes V Weil A Integrative medicine and systemic outcomes research: issues in the emergence of a new model for primary health care Arch Int Med 2002 162 2 133 10.1001/archinte.162.2.133 11802746

[B30] Ritenbaugh C Verhoef M Fleishman S Boon H Leis A Whole systems research: a discipline for studying complementary and alternative medicine Altern Ther Health Med 2003 9 4 32 12868250

[B31] Bond GR Evans L Salyers MP Williams J Kim HW Measurement of fidelity in psychiatric rehabilitation Ment Health Serv Res 2000 2 75 87 10.1023/A:1010153020697 11256719

[B32] Sechrest L West SG Phillips MA Redner R Yeaton W Some neglected problems in evaluation research: strength and integrity of treatments Evaluation Studies Review Annual 1979 4 15 35

[B33] Woolf SH Johnson RE Inattention to the fidelity of health care delivery is costing lives Am J Public Health 2007 97 10 1732 17761559 10.2105/AJPH.2007.116707 PMC1994195

[B34] Adler SR Integrative medicine and culture: toward an Anthropology of CAM Med Anthro Quart 2002 16 4 412 10.1525/maq.2002.16.4.412 PMC2764405 12500614

[B35] Long AF Outcome measurement in complementary and alternative medicine: unpicking the effects J Altern Complement Med 2002 8 6 777 786 10.1089/10755530260511793 12614531

[B36] Coulter ID Khorsan R Magnabosco JL, Manderscheid RW Complementary alternative and integrative medicine: current challenges for outcomes measurement Outcome Measurement in the Human Services 2011 Washington, DC: NASW Press 163 178

[B37] Federal Coordinating Council for Comparative Effectiveness Research Report to the President and the Congress 2009 Washington, DC: Federal Coordinating Council for Comparative Effectiveness Research

[B38] Benson K Hartz AJ A comparison of observational studies and randomized, controlled trials New England J Med 2000 342 25 1878 1886 10.1056/NEJM200006223422506 10861324

[B39] Concato J Shah N Horwitz RI Randomized, controlled trials, observational studies, and the hierarchy of research designs New England J Med 2000 342 25 1887 1892 10.1056/NEJM200006223422507 10861325 PMC1557642

[B40] Heinsman DT Shadish WR Assignment methods in experimentation: When do nonrandomized experiments approximate answers from randomized experiments? Psychological Methods 1996 1 2 154

[B41] Tunis SR Benner J McClellan M Comparative effectiveness research: policy context, methods development and research infrastructure Statistics Med 2010 29 19 1963 1976 10.1002/sim.3818 20564311

[B42] Teutsch SM Berger ML Weinstein MC Comparative effectiveness: asking the right questions, choosing the right method Health Affairs 2005 24 1 128 132 10.1377/hlthaff.24.1.128 15647223

[B43] Chokshi DA Avorn J Kesselheim AS Designing comparative effectiveness research on prescription drugs: lessons from the clinical trial literature Health Affairs 2010 29 10 1842 1848 10.1377/hlthaff.2010.0843 20921484

[B44] Maizes V Caspi O The principles and challenges of integrative medicine Western J Med 1999 171 3 148 PMC1305791 10560281

[B45] Committee on the Future of Primary Care Donaldson MS, Yordy KD, Lohr KN, Vanselow NA Primary Care: America's Health in a New Era 1996 Washington, DC: Institute of Medicine 25121221

[B46] The defining principles of integrative medicine [http://integrativemedicine.arizona.edu/about/definition.html]

[B47] Joint Principles of the Patient-Centered Medical Home [http://www.pcpcc.net/content/joint-principles-patient-centered-medical-home] 10.2169/naika.104.141 26571790

[B48] Dodds SE Herman PM Sechrest L Abraham I Logue MD Grizzle AL Rehfeld RA Urbine TJ Horwitz R Crocker RL Maizes VH When a whole practice model is the intervention: developing fidelity evaluation components using program theory-driven science for an integrative medicine primary care clinic Evidence-Based Complementary and Alternative Medicine 2013 2013 Article ID 652047 11 10.1155/2013/652047 PMC3863495 24371464

[B49] Caliendo M Kopeinig S Some practical guidance for the implementation of propensity score matching J Econ Surveys 2008 22 1 31 72 10.1111/j.1467-6419.2007.00527.x

[B50] Adult Visit Questionnaire 2.0 [http://cahps.ahrq.gov/clinician_group/]

[B51] Behavioral Risk Factor Surveillance System Survey Questionnaire [http://www.cdc.gov/brfss]

[B52] Hibbard JH Stockard J Mahoney ER Tusler M Development of the Patient Activation Measure (PAM): conceptualizing and measuring activation in patients and consumers Health Services Res 2004 39 4p1 1005 1026 10.1111/j.1475-6773.2004.00269.x PMC1361049 15230939

[B53] Hibbard JH Mahoney ER Stockard J Tusler M Development and testing of a short form of the patient activation measure Health Services Res 2005 40 6p1 1918 1930 10.1111/j.1475-6773.2005.00438.x PMC1361231 16336556

[B54] Cohen S Kamarck T Mermelstein R A global measure of perceived stress J Health Social Behavior 1983 24 385 396 10.2307/2136404 6668417

[B55] Littman AJ White E Satia JA Bowen DJ Kristal AR Reliability and validity of 2 single-item measures of psychosocial stress Epidemiology 2006 17 4 398 403 10.1097/01.ede.0000219721.89552.51 16641618

[B56] Buysse DJ Reynolds CF Monk TH Berman SR Kupfer DJ The Pittsburgh Sleep Quality Index: a new instrument for psychiatric practice and research Psychiatry Res 1989 28 2 193 213 10.1016/0165-1781(89)90047-4 2748771

[B57] Hargraves JL Hays RD Cleary PD Psychometric properties of the consumer assessment of health plans study (CAHPS®) 2.0 adult core survey Health Services Res 2003 38 6p1 1509 1528 10.1111/j.1475-6773.2003.00190.x PMC1360961 14727785

[B58] Huskisson E Measurement of pain The Lancet 1974 304 7889 1127 1131 10.1016/S0140-6736(74)90884-8 4139420

[B59] Krupp LB LaRocca NG Muir-Nash J Steinberg AD The fatigue severity scale: application to patients with multiple sclerosis and systemic lupus erythematosus Archives of neurology 1989 46 10 1121 10.1001/archneur.1989.00520460115022 2803071

[B60] Kroenke K Spitzer RL Williams JBW The Patient Health Questionnaire-2: validity of a two-item depression screener Medical Care 2003 41 11 1284 1292 10.1097/01.MLR.0000093487.78664.3C 14583691

[B61] Kroenke K Spitzer RL Williams J Monahan PO Löwe B Anxiety disorders in primary care: prevalence, impairment, comorbidity, and detection Ann Int Med 2007 146 5 317 10.7326/0003-4819-146-5-200703060-00004 17339617

[B62] Ware JE Jr Kosinski M Keller SD A 12-Item short-form health survey: construction of scales and preliminary tests of reliability and validity Medical Care 1996 34 3 220 233 10.1097/00005650-199603000-00003 8628042

[B63] Bech P Olsen LR Kjoller M Rasmussen NK Measuring well‒being rather than the absence of distress symptoms: a comparison of the SF‒36 Mental Health subscale and the WHO‒Five well‒being scale Int J Methods Psychiatric Res 2003 12 2 85 91 10.1002/mpr.145 PMC6878541 12830302

[B64] Reilly MC Zbrozek AS Dukes EM The validity and reproducibility of a work productivity and activity impairment instrument Pharmacoeconomics 1993 4 353 353 10.2165/00019053-199304050-00006 10146874

[B65] Ritter PL Stewart AL Kaymaz H Sobel DS Block DA Lorig KR Self-reports of health care utilization compared to provider records J Clin Epidemiol 2001 54 2 136 141 10.1016/S0895-4356(00)00261-4 11166528

[B66] Bennett RM Friend R Jones KD Ward R Han BK Ross RL The revised Fibromyalgia Impact Questionnaire (FIQR): validation and psychometric properties Arthritis Res Ther 2009 11 4 120 10.1186/ar2734 19664287 PMC2745803

[B67] Kroenke K Spitzer RL The PHQ-9: a new depression diagnostic and severity measure Psychiatric Annals 2002 32 9 509 515

[B68] Kroenke K Spitzer RL Williams JBW The PHQ‒9 J Gen Int Med 2001 16 9 606 613 10.1046/j.1525-1497.2001.016009606.x PMC1495268 11556941

[B69] Spitzer RL Kroenke K Williams JBW Lowe B A brief measure for assessing generalized anxiety disorder: the GAD-7 Arch Int Med 2006 166 10 1092 10.1001/archinte.166.10.1092 16717171

[B70] D’Agostino RB Sr Vasan RS Pencina MJ Wolf PA Cobain M Massaro JM Kannel WB General cardiovascular risk profile for use in primary care Circulation 2008 117 6 743 753 10.1161/CIRCULATIONAHA.107.699579 18212285

[B71] Anderson KM Odell PM Wilson PWF Kannel WB Cardiovascular disease risk profiles American Heart J 1991 121 1 293 298 10.1016/0002-8703(91)90861-b 1985385

[B72] Grundy SM Cleeman JI Daniels SR Donato KA Eckel RH Franklin BA Gordon DJ Krauss RM Savage PJ Smith SC Jr Diagnosis and management of the metabolic syndrome: an American Heart Association/National Heart, Lung, and Blood Institute scientific statement Curr Opinion Cardiol 2006 21 1 1 10.1097/01.hco.0000200416.65370.a0 16355022

[B73] Safran DG Karp M Coltin K Chang H Li A Ogren J Rogers WH Measuring patients' experiences with individual primary care physicians J Gen Int Med 2006 21 1 13 21 10.1111/j.1525-1497.2005.00311.x PMC1484603 16423118

[B74] Mercer SW Maxwell M Heaney D Watt GCM The consultation and relational empathy (CARE) measure: development and preliminary validation and reliability of an empathy-based consultation process measure Family Practice 2004 21 6 699 705 10.1093/fampra/cmh621 15528286

[B75] Loo R Loewen P A confirmatory factor-analytic and psychometric examination of the team climate inventory full and short versions Small Group Research 2002 33 2 254 265 10.1177/104649640203300205

[B76] Rohland BM Kruse GR Rohrer JE Validation of a single-item measure of burnout against the Maslach Burnout Inventory among physicians Stress and Health 2004 20 2 75 79

[B77] National Center for Health Statistics Ambulatory Health Care Data 2012 Centers for Disease Control and Prevention

[B78] Brazier JE Roberts J The estimation of a preference-based measure of health from the SF-12 Medical Care 2004 42 9 851 859 10.1097/01.mlr.0000135827.18610.0d 15319610

[B79] Cohen J Statistical Power Analysis for the Behavioral Sciences 1988 2 Hillsdale, New Jersey: Lawrence Erlbaum Associates

[B80] Heo M Faith MS Mott JW Gorman BS Redden DT Allison DB Hierarchical linear models for the development of growth curves: an example with body mass index in overweight/obese adults Stat Med 1911–1942 2003 22 10.1002/sim.1218 12754724

[B81] Raudenbush SW Bryk AS Hierarchical Linear Models: Applications and Data Analysis Methods, Second Edition edn 2002 Thousand Oaks, CA: Sage Publications

[B82] Singer JD Using SAS PROC MIXED to fit multilevel models, hierarchical models, and individual growth curve models J Educ Behav Stat 1998 24 4 323 355

[B83] Barber JA Thompson SG Analysis of cost data in randomized trials: an application of the non-parametric bootstrap Stat Med 2000 19 3219 3236 10.1002/1097-0258(20001215)19:23<3219::AID-SIM623>3.0.CO;2-P 11113956

[B84] Efron B Tibshirani RJ An Introduction to the Bootstrap 1994 Champman & Hall/CRC: Boca Raton

[B85] Von Elm E Altman DG Egger M Pocock SJ Gøtzsche PC Vandenbroucke JP The Strengthening the Reporting of Observational Studies in Epidemiology (STROBE) statement: guidelines for reporting observational studies Prevent Med 2007 45 4 247 251 10.1016/j.ypmed.2007.08.012 17950122

